# Salmagundi

**Published:** 1884-02

**Authors:** 


### SALMAGUNDI.

THE Russian for "dentist" is "dantajowwitchchefchew." The word is expressive, for it is itself a jaw-breaker.

ONIONS are recommended for persons of studious and sedentary habits, but an hour's airing each day will do great good.

IN the cities of Brazil where coffee is largely used as a beverage, alcoholism is unknown. The Cincinnati *Price Current* says coffee is "nearly as good as Cincinnati beer."—*Daily Paper.*

PROF. A. F. A. KING, writing in a recent issue of *Popular Science*, charges mosquitoes with being "the most active and efficient agents in the dessemination of malarial poison."

OUR good friend, Dr. F. W. Sage, received a holiday gift from his estimable wife during the Christmas week, in the shape of a bouncing boy. Dr. S. has our hearty congratulations.

DR. FRANK HUNTER tells us that he is prepared to supply his "Champion Nerve Paste" in any quantity; the formula for which he kindly gave to the profession at the last meeting of the M. V. D. A.

IT has been discovered from statistics that in Paris the number of typhoid fever cases increases after each heavy rainfall. It is not difficult, therefore, to trace the trouble to the stirring up and setting free of foul sewer gases.

SAID a Baltimore fakir, as he pulled up stakes and started home on a cold day: "What with the women who jerk teeth, and the men who play the banjo, and the snides who tell fortunes, the old legitimate tooth paste venders haven't much show."

NEW YORK CITY has about forty public and private hospitals, with more than 7,000 beds. The fourteen hospitals which are supported chiefly by the public, can accommodate 5,000 patients.

Messrs. Spencer & Crocker have devised a method for attaching a "Post" student lamp to the socket of the automatic bracket, and have furnished it with a large bulls-eye, which sends a flood of rich light into the mouth, thus enabling the operator to take advantage of time that would otherwise be lost during the murky weather of the winter.

---

### GOOD TEETH AND GOOD INTELLECT.

The recent discussion in the French medical journals on the relation of the teeth to the brain, and their conclusions, are of importance to all brain-workers. Dr. Championniere recommends that parents and guardians should pay close attention to the condition of the teeth of those under their care, and should, when any signs of premature decay are noticeable, give their charges a holiday.—*Medical and Surgical Reporter.*

---

Some of our newspapers express a fear that the United States is rapidly becoming a dumping ground not for the pauper and criminals of Europe alone, but for the insane as well. The ratio of insanity in this country is increasing at a terrible rate. In 1850 there was one insane in 1,545 of native population, and one in 1,095 of alien population. In 1880 there was one insane in 662 of native population, and one in 250 of alien population.—*Cincinnati Commercial Gazette.*

---

We are very much pleased to see "Caulk's Annual" for 1883–84, and heartily commend the effort to gather statistical information concerning dental matters. The list embraces the number of dentists, dealers, etc., colleges, necrology, patients, societies, and periodicals, all of which cannot fail to be of service. Let all dentists, through their societies, collect matter and send it to Dr. Caulk, so that the "Annual" may annually increase in value.

---

"A New York dentist says that, as a general rule, most of the cavities in the teeth commence to form before the individual is twenty years of age, especially if habits are 'too fast,' and it is seldom that new cavities appear after thirty. If the teeth are carefully watched and filled until the latter age is reached there is good chance that they will last a lifetime if properly cared for. Gold filling is not always the best. Amalgam fillings frequently prove the most durable and serviceable."

The last two sentences show pretty clearly what kind of a man that " New York Dentist " is and how he seeks to give himself a name for sake of " biz " you know, by being interviewed.

---

### NOT A BAD IDEA.

Dr. C. F. Naismith thus writes to the *Lancet*, October 27, 1883 : "It is quite a common occurrence for young mothers to complain of the rapid decay of their teeth. This is no doubt caused by the abstraction of lime from the mother's blood to form the bones of the fœtus. Hence the craving for chalk and kindred substances. How would it do to prescribe phosphate and carbonate of lime regularly for pregnant females, to make up the deficiency ? "

It would, very likely, make good teeth and bones for the child, and help the mother's, but would also, in all probability, greatly increase the difficulty of parturition, as Dr. Wm. Van Antwerp once discovered.

---

### HOW FAR ARE DENTISTS DOCTORS?

A curious legal question has come recently before the courts in Berlin. A young woman called on a dentist to have some teeth extracted. He told her that the operation would not be prudent in certain conditions of her system, and with her consent made a manual examination to ascertain her condition as to pregnancy, etc. Her family brought suit against him. The court of first instance acquitted him on the ground that he had acted within the proper limits of his profession ; the higher court, however, reversed this decision, and sentenced him to eight weeks' imprisonment, stating that as a dentist he was not called upon to make such an examination, and that it was from improper motives.—*Medical and Surgical Reporter.*

"SEARCH for the truth is the noblest occupation of man; its publication a duty," wrote Madame Stael; Orville Dewey a little more forcibly remarks that "withholding facts is robbery." From both of these we infer that knowledge is the common heritage of all mankind and that the individual who by chance or purpose becomes possessed of anything likely to benefit his fellow men, is, in honor bound, charged to communicate it to the world. The printing press offers the widest facilities for the dissemination of truth, reaching all those who seek to better their condition and by their increased capability to render their sphere in life more valuable to the common weal.

With these hints to stimulate contribution, we hope that our readers will all send us something for the department of *Salmagundi.*

---

DENTAL STATISTICS, BY A. H. THOMPSON, D.D.S., TOPEKA, KAN.

One great need in the scientific study of dental subjects of all kinds is the need of reliable and complete statistics. Especially is this true in relation to the occurrence of dental diseases; their comparative frequency, the peculiarities of their surroundings as modifying their malignancy, etc. An example of their value is furnished by Magitot's study of the statistics of decay, as furnished by the examiner's reports of its frequency in the French army. By it Magitot was enabled to formulate tables, and a map, showing the geograpical distribution of dental caries in France, and its variable occurrence in different races. He illustrated that caries is probably due more to miscegenation than to all other pre-disposing causes combined. But these tables were imperfect and incomplete, yet, so far as they go, are invaluble. They demonstrate what incalculable results might be obtained from tables prepared from statistics intelligently collected, and reliably collated. We need such statistics to gain a scientific knowledge of caries, if not indeed to inform us as to the simple cause of caries, which is yet in dispute. The most intelligent effort yet made to secure such statistics, is the step taken by the Illinois State Dental Society. The progressive brethren there have agreed to distribute blanks for dentists and other qualified persons to fill up and return to a committee, who will collate them and prepare tables. It is intended to examine the teeth of the children of this great land, as they are found in the public and private institutions, schools, etc., and collect data relative to size, quality, and health of teeth, irregularity, caries, and other diseases, and the physical condition of the child, health, occupation, race, nativity, etc., etc. The value of such statistics can be at once perceived. They would, for instance, give us a fair idea of the peculiarites of organization, surroundings, etc., of caries, so that we could estimate the active causes of this scourge, and begin to apply preventive measures with some hope of success. This one instance, showing the value of statistics, should be all the argument necessary to convince men of the need of inaugurating the enterprise of collecting them without delay.

WE reprint this brief article of Dr. Thompson's from "Caulk's Annual," because it alludes to a subject that is of vast importance, and one that in the future will employ much of the time and brains of the profession. For more detailed treatment of the idea, the reader is referred to the paper on the "Geographical Distribution of Dental Caries in the United States" in the October number of the Ohio *State Journal* for 1881.

